# Draft Genome Sequence of Methicillin-Resistant *Staphylococcus aureus* KT/Y21, a Sequence Type 772 (ST772) Strain Isolated from a Pediatric Blood Sample in Terengganu, Malaysia

**DOI:** 10.1128/genomeA.00271-14

**Published:** 2014-04-10

**Authors:** Zarizal Suhaili, Soo-Sum Lean, Azifah Yahya, Mohd Nasir Mohd Desa, Abdul Manaf Ali, Chew Chieng Yeo

**Affiliations:** a Faculty of Agriculture, Biotechnology and Food Sciences, Universiti Sultan Zainal Abidin, Gong Badak Campus, Terengganu, Malaysia; b Institute of Biological Sciences, Faculty of Science, Universiti Malaya, Kuala Lumpur, Malaysia; c Department of Biomedical Science, Faculty of Medicine and Health Sciences, Universiti Putra Malaysia, Serdang, Selangor, Malaysia; d Faculty of Medicine and Health Sciences, Universiti Sultan Zainal Abidin, City Campus, Kuala Terengganu, Terengganu, Malaysia

## Abstract

Here, we report the draft genome sequence of a methicillin-resistant *Staphylococcus aureus* (MRSA) strain, KT/Y21, isolated from a blood sample of a pediatric patient. This strain belongs to sequence type 772 (ST772), harbors the staphylococcal cassette chromosome *mec* element (SCC*mec*) type V, and is positive for the Panton-Valentine leukocidin (PVL) pathogenic determinant.

## GENOME ANNOUNCEMENT

*Staphylococcus aureus* is an important human pathogen equipped with an arsenal of virulence and antimicrobial resistance genes (1), thus making it one of the leading pathogens causing bloodstream infections in the hospital setting and within the community. Some *S. aureus* strains produce Panton-Valentine leukocidin (PVL), a bicomponent cytotoxin encoded in the PVL prophage that is made up of two protein components designated Luk-S-PV and Luk-F-PV, which function to destroy leukocytes by creating pores in the cell membranes (2). *S. aureus* strain KT/Y21 was isolated from a blood sample from a newborn patient with septicemia in the pediatric intensive care unit (PICU) of the main tertiary referral hospital in Kuala Terengganu, Malaysia. Phenotypic identification was confirmed by a MicroLog system (version 4.2) (Biolog, Inc., Hayward, CA), and antimicrobial susceptibility testing profiles indicated that strain KT/Y21 is multidrug resistant, with resistances toward cefoxitin, ciprofloxacin, fusidic acid, gentamicin, kanamycin, mupirocin, neomycin, oxacillin, penicillin, and tobramycin.

Genome sequencing of *S. aureus* KT/Y21 was performed using the Illumina Genome Analyzer IIx with 100-bp paired-end reads. The paired-end reads were trimmed and assembled *de novo* using CLC Genomics Workbench 5.1 (CLC bio, Denmark). The draft genome was annotated using Blast2GO 2.5.0 (3) and subsequently validated using Rapid Annotation Subsystems Technology (RAST) (4) and the Bacterial Annotation System (BASys) (5). Multilocus sequence typing (MLST) was performed using a local BLAST search to identify the seven gene loci (i.e., *arcC*, *aroE*, *glpF*, *gmk*, *pta*, *tpi*, and *yqiL*) as described in Enright et al. (6). The *spa* type was assigned using DNAGear (7).

Altogether, 70 contigs were obtained from the *de novo* assembly, with an accumulated length of 2,763,469 bp and a G+C content of 32.6%. A total of 2,569 coding sequences (CDSs) were identified and annotated. Of the CDSs, 4.2% were associated with the cell wall and capsule, 3.5% are associated with virulence, disease, and defense mechanisms, and 2.9% are related to the stress response, which contributes to adaptation in the host and survivability. Meanwhile, 3.19% are related to membrane transport, part of which is believed to contribute to the antibiotic resistance mechanism of the strain. Genotypically, *S. aureus* KT/Y21 is of the sequence type 772 (ST772) lineage, *spa* type t657, and *agr*II and *dru* type dt10ao. KT/Y21 also harbors the PVL pathogenic genes. Clinical isolates of *S. aureus* of the same ST772 lineage and that are PVL positive have been reported in Malaysia (8). These isolates might have originated from Bangladesh due to similarities with the so-called Bengal Bay Clone ST772 lineage (9, 10), as there are many Bangladeshis working as laborers and contract workers in Malaysia.

The *S. aureus* KT/Y21 genome also contains genomic islands, such as the staphylococcal cassette chromosome *mec* element (SCC*mec*) with a type V *ccrC* complex, the νSaα and νSaβ pathogenicity islands, and the PVL prophage (11). However, no plasmids were identified from the draft genome sequence. Sequencing *S. aureus* KT/Y21 has generated the first draft genome of an ST772 lineage from Malaysia, thus providing a reference for future comparative genomics of this important pathogen.

### Nucleotide sequence accession numbers.

This whole-genome draft sequence project has been deposited at DDBJ/EMBL/GenBank under the accession no. AOCQ00000000. The version of the genome described in this paper is the first version (GenBank accession no. AOCQ01000000).
